# Supplementary material for: Differentiation of Human Cardiac Atrial Appendage Stem Cells into Adult Cardiomyocytes: A Role for the Wnt Pathway?
Source: Int J Mol Sci. 2020 May 30;21(11):3931. doi: 10.3390/ijms21113931 (PMC7312541; doi:10.3390/ijms21113931)
Supplement: Supplementary file 1 [file ijms-21-03931-s001.pdf]

SUPPLEMENTARY MATERIAL

RAA

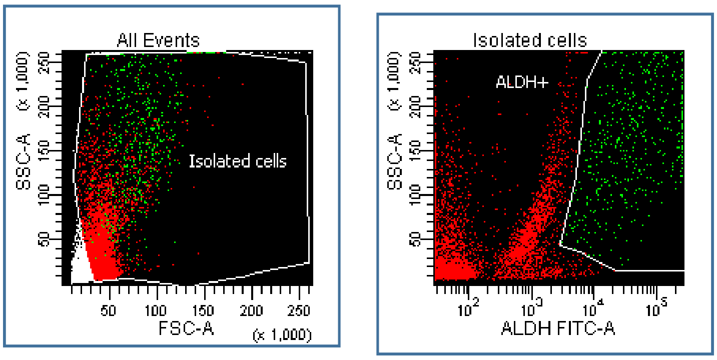

LAA

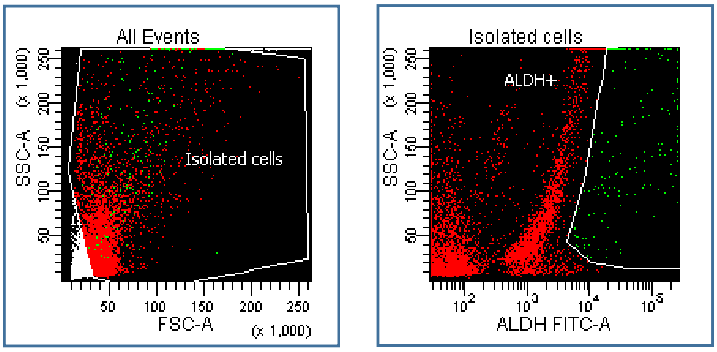

Figure 1. Percentages of ALDH^{br} cells in the human atrial appendage. Representative FACS plots showing the percentage of ALDH^{br} cells in right (top) and left atrial appendage (bottom). LAA = left atrial appendage; RAA = right atrial appendage.

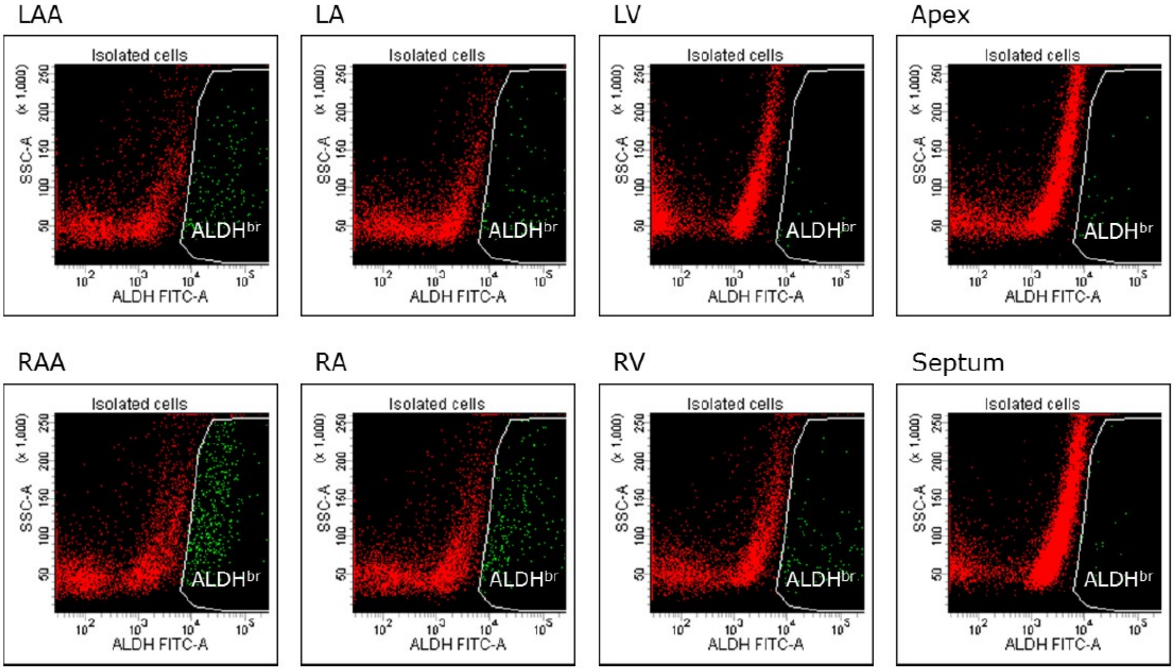

Figure 2. Percentages of ALDH^{br} cells in different compartments of the pig heart. Representative FACS plots showing the percentage of ALDHbr cells in different compartments of the pig heart. LA = left atrium; LAA = left atrial appendage; LV = left ventricle; RA = right atrium; RAA = right atrial appendage; RV = right ventricle.
